# Intersectional dehumanization in faces: Facial attractiveness and perceived intelligence differentially predict dehumanization across intersecting race and gender identities

**DOI:** 10.1371/journal.pone.0351086

**Published:** 2026-07-01

**Authors:** Neelamberi Devi Klein, Kurt Hugenberg

**Affiliations:** Department of Psychology and Brain Sciences, Indiana University Bloomington, Bloomington, Indiana, United states of America; Yeditepe University, TÜRKIYE

## Abstract

Past work has found that more attractive and more intelligent appearing faces are ascribed more humanlike sophistication at zero acquaintance, but past work has only employed White faces. Of interest in the present work is whether similar effects occur across target race. Across two studies we investigate if the relationships between attractiveness and perceived intelligence vary across Black and White, male and female faces. Across both studies, we find that more attractive faces are evaluated as more humanlike across target race and gender, but notably that target race moderates the predictiveness of perceived intelligence. Perceived intelligence only relates to humanness for White targets, but not Black. These findings provide support for the theory that face trait relationships are often dependent on target identities, such as race and are consistent with extended research indicating that Black Americans’ minds may be devalued.

## Introduction

Ascribing huma‌‌nlike capacities to or withholding humanlike capacities from our fellow humans has long been of interest to psychologists, in part because of the important role it plays in how others are treated. Withholding humanity from others excludes them from the moral community facilitating harmful treatment [1–3]. Although dehumanization is separable from prejudice [4], dehumanization can trigger discrimination and aggression [5–7]. and often co-occurs with disgust and contempt [8,9]. However, one consistent finding is that when humanity is withheld from others, dehumanized persons are not ascribed the full human range of emotions [8,10], cognitive faculties, and moral faculties [6,9,11].

Past work has demonstrated that both *group*-level beliefs (e.g., belonging to a culturally devalued group) and *individual*-level perceptual cues (e.g., whether a target’s face appears attractive or intelligent) can influence the extent to which people ascribe humanness to others [10,12]. In one powerful example of group-level beliefs on dehumanization, Goff and colleagues [13] demonstrated that White Americans often associate Black people with apes, and this Black-ape association has powerful implications for behavior. In an example of the effects of individual-level perceptual cues, Alaei and colleagues [12] found that White target faces appearing more attractive or more intelligent were rated as more humanlike than their less attractive and/or less intelligent-appearing counterparts.

Preliminary evidence also indicates that these group-based and individual-target based cues can work in tandem to drive dehumanization. Specifically, in Alaei and colleagues’ [12] work both White men’s and White women’s faces were judged as more humanlike when they appeared more attractive and intelligent. However, the strength of the attractiveness-humanness and perceived intelligence-humanness links differed across target gender. The attractiveness-humanness relationship was stronger for female than male targets, and the perceived intelligence-humanness relationship was stronger for male than female targets. These gendered effects were especially true for perceivers who more strongly held traditional gender stereotypes. In line with other recent findings, Alaei and colleagues’ [12] research suggests that trait relationships differ for different groups; in this case, relationships between attractiveness and humanness and between perceived intelligence and humanness differ for White male and White female targets [14,15].

However, to our knowledge no work has yet explored whether these (de)humanizing effects of attractiveness and perceived intelligence occur equivalently across target race and target gender intersections. We address just this question in the present work, focusing specifically on male and female, Black and White targets. We see this as both a theoretically novel and practically important question. First, Black people are a chronically dehumanized group, which allows us to investigate whether the individual-level perceptual cues linked to humanness continue as robust predictors in a group that is often powerfully dehumanized, and whether both attractiveness and apparent intelligence have similar effects across target race. Second, as we outline below, whereas past work has shown that both attractiveness and perceived intelligence predict humanness ascriptions, specific racial stereotypes suggest that Black people are often valued for their bodies rather than their minds [9,16]. Thus, it is unclear whether there may be asymmetric effects of facial attractiveness and perceived intelligence across White and Black people. The present work can test the boundary conditions of these previously established effects of perceptual cue-based dehumanization, while also extending our understanding of the dehumanizing treatment of Black people, an important goal in its own right.

### How might dehumanization by facial attractiveness and apparent intelligence differ across race?

Dehumanization is historically and unequally applied to Black people compared to White people in America. Dehumanization of Black people has often taken the form of viewing them in terms of bodies more than minds. Long standing stereotypes show the tendency to perceive Black individuals as less intelligent, more animalistic, and lacking impulse control [13,17], while stereotyping Black men as physically large and Black women as sexually promiscuous [16,18,19]. Indeed, even within groups such as politicians, negative racial attitudes are related to Black politicians being perceived more negatively than White politicians for the same scandal [20]. Thus, insofar as Americans tend to dehumanize Black people by emphasizing their bodies (size; sexuality) and minimizing their minds (unintelligent; unregulated), this may have implications for which perceptual cues perceivers use to infer humanness across target race and target gender.

In addition to racial differences in dehumanization, there is evidence suggesting that perceived attractiveness, a perceptual predictor of dehumanization, is both racialized and gendered. Images of both Black men and women put through a beautifying filter were perceived as less Black-prototypical, and Black women’s photos were perceived as more White-prototypical compared to the original photos, indicating that attractiveness of faces is influenced by the degree that each face is perceived as prototypically White and Black [21]. Among mixed-race women’s faces, faces perceived as more White-prototypical were perceived as more attractive, whereas faces perceived as more Black-prototypical were perceived as more attractive amongst male faces [22], again suggesting that racial and gendered cues are linked to attractiveness judgments.

The premise ‌‌that the effects of attractiveness and perceived intelligence may be different at specific intersections of race and gender (e.g., Black women) is consistent with recent models of intersectional stereotyping. Indeed, such intersectional stereotyping is of increasing interest in social psychology, resulting in a variety of theoretical frameworks to integrate findings [23]. As we note above, recent research indicates that relationships between apparent traits differ across different race x gender intersectional groups. Indeed, Stolier and colleagues [14] argue that the relationship between traits (e.g., how strongly attractiveness and humanness correlate) is a product of learning, and further recent work has shown that these relationships can differ across race x gender intersectional identities. Supporting this, Xie and colleagues [15] had participants rate race x gender intersectional groups on various traits (i.e., to what extent are Asian men aggressive, intelligent, attractive, etc.) and had participants rate faces of various intersectional identities (e.g., Asian men) on those same traits. They found that the relationship between stereotypic traits in a group predicted the relationship between trait ratings in faces of that group. Put simply, trait relationships differ across different race x gender intersectional identities. This is important for the present work because we seek to investigate how relationships between different traits (here, between facial attractiveness and humanness, and between apparent intelligence and humanness) differ across race x gender intersections. Whereas Alaei and colleagues [12] found facial attractiveness and perceived intelligence to be related to dehumanization for White men and women, of interest is whether these results differ for Black men and women.

### Current research

The present work aims to replicate and extend Alaei and colleagues [12] by investigating the relationship between perceived traits (perceived attractiveness and perceived intelligence) and ascriptions of humanness across target race (Black and White) and target gender (men and women) intersections. Following Alaei and colleagues’ work, our two studies adopted a by-stimulus approach, measuring how variability in stimulus-level attractiveness and apparent intelligence predicts variability in stimulus-level ascriptions of humanness.

Across two studies conducted between 17^th^ November 2022 and 17^th^ September 2025, naïve U.S. participants rated 360 faces from the Chicago Face Database [24]. Participants were randomly assigned to rate facial attractiveness, or perceived intelligence, or Blatant Dehumanization of a large number of faces. As target age and gaze direction is implicated in trait perception, all stimuli were between 18–40 years old and had forward directed gazed [25,26]. In Study 1, participants rated either Black men, Black women, White men, or White women on a single trait. In Study 2, participants rated a subset of all four race-gender intersections on a single trait. By-stimulus analyses were conducted to assess how perceived attractiveness and perceived intelligence of a given face predicted the ascribed humanness of that face. We expected to replicate the findings of Alaei and colleagues [12] that both attractiveness and perceived intelligence would predict ascriptions of humanness for White faces, and that attractiveness would be the strongest predictor of ascriptions of humanness for White women, whereas perceived intelligence would be the strongest predictor for White men.

Of primary interest, however, was what would occur for Black women and men. Multiple possibilities seemed plausible *a priori*. First, in line with recent work showing differing stereotypic trait relationships across target race x target gender intersections, it seemed plausible that White faces, and especially White men’s faces, might show a stronger perceived intelligence-humanness link than the other intersections. Indeed, consistent with Alaei and colleagues’ original findings showing that intelligent appearing White men were seen as especially human and given the substantial stereotypes that minimize Black people’s minds, this might lead to an especially weak link between perceived intelligence and humanness for Black people. Second, it seemed plausible that there may be an especially strong link between attractiveness and humanness for White women and/or for Black men. Given past work showing a link between racial prototypicality and attractiveness for both White women and for Black men [22], this suggests that both groups may be especially valued for their physical attractiveness. Finally, it was also plausible *a priori* that gender stereotypes would continue to drive judgments for both White and Black targets equally, replicating Alaei and colleagues across both target race conditions.

### Study 1

In Study 1, participants rated the attractiveness, the perceived intelligence, or the humanness of Black women, Black men, White women, or White men (target race x target gender manipulated between-subjects). Of interest was whether the past links between attractiveness and humanness (especially for White female faces) and intelligence and humanness (especially for White male faces) would replicate for Black female and male targets.

### Method

#### Participants.

Sample size was determined utilizing Westfall and colleagues [27] “both-within-condition” designs. Contour plots demonstrate that with 256 stimuli and 256 participants, studies would be at 99% power to detect an effect of *d* = 0.5. Similarly, at 80% power, the same number of stimuli and participants would be powered to detect effects between 0.2–0.3. Thus, as our stimuli sample was held at 360, we aimed for a final sample around 256. Additional participants were recruited to account for participant exclusion. Participants (*n* = 423) were mTurk workers paid $3.62 for a 30-minute study recruited to take part in the study. 64 participants were removed for having given homogeneous responses or not finishing the study, and 22 participants were removed for failing to report taking the study seriously, resulting in an analytic sample of *n* = 337. Due to an experimenter error in the wording of the Black women Dehumanization condition, this condition was dropped from the original data collection and recollected separately on CloudResearch between September 9^th^ and September 17^th^ of 2025. Participants were U.S. residents and fluent in English. 48.1% of participants identified as female, 48.1% as male, 3.8% identifying as transgender/non-conforming or not reporting a gender. Mean age was 39.63 (*SD* = 12.02). Participants primarily identified as White (68.2%), but a notable portion identified as Black (11.9%) or selected multiple racial identities (9.5%). We did not screen participants by race, both because we did not have specific participant-level hypotheses and because this allowed us to have a more diverse sample. Many participants identified as slightly to very liberal in political identity 49.6%, with 16.6% identifying as moderate, and 32.0% identifying as slightly to very conservative (6 participant did not report a listed political ideology).

### Stimuli & Measures

**Face Stimuli.** Stimuli were 360 neutral expression faces from the Chicago Face Database [24]. We randomly selected 90 targets from each target race x target gender intersection. Faces ranged between 18–40 years of age and were all forward faces with direct gaze.

**Blatant Dehumanization** Ascriptions of humanness were assessed using the Blatant Dehumanization scale); see Online Supplement for complete wording [4].

**Perceived Traits** Following Alaei and colleagues, perceived attractiveness and perceived intelligence were assessed using a 7-point Likert scale (1 = Not at all attractive[intelligent] to 7 = Extremely attractive[intelligent]); see Online Supplement for complete wording.

### Procedure

All procedures and materials were approved by the Institutional Review Board at Indiana University Bloomington. After providing written informed consent, participants were randomly assigned to rate all 90 faces from a single target race x target gender condition (e.g., Black women) in a random order on a single trait (e.g., attractiveness). After finishing their ratings, participants were thanked, debriefed, and paid for their time.

## Results and discussion

### Analytic strategy

Analyses were conducted at the stimulus-level. Thus, mean ratings for each trait were computed for each target face, allowing us to investigate whether variability in attractiveness and perceived intelligence predict ascriptions of humanness for target race x target gender groups. The method of data collection was to have a given participant only evaluate the faces on one dimension (attractiveness or perceived intelligence or humanness). Although this ensures that participants are not aware of the specific hypotheses about the relationship between the variables, it does preclude a multilevel modeling approach.

Instead, given that this work was a close conceptual replication and extension of Alaei and colleagues, we followed their analytic strategy closely. Alaei and colleagues predicted ascriptions of humanness from perceived attractiveness of target, perceived intelligence of target, target gender, target gender x perceived attractiveness, and target gender x perceived intelligence. In our analyses we added those same predictors as well as target race and target race’s interaction with those extant factors (i.e., target race x target gender, target race x perceived attractiveness, target race x perceived intelligence, target gender x target race x perceived attractiveness, and target gender x target race x perceived intelligence). Prior to being input into the model, all trait evaluations were Z-scored. Target gender and target race were effects coded such that for target gender 1 = Female and −1 = Male, and for target race 1 = Black and −1 = White.

### Analyses and Discussion

Our overall model predicting dehumanization from perceived traits, as well and target race and gender was significant, *F*(11, 348) = 111.20, *p* < .001, *R*^*2*^ = .77. As seen in Table 1, we replicate Alaei’s basic finding that both facial attractiveness and perceived intelligence predicted ascriptions of humanness, although notably we do not observe that these effects vary by target gender. The perceived intelligence-to-humanness link however, does vary by target race as indicated by the race x perceived intelligence interaction, *β* = −0.12, *t* = −2.84, *p* = .004. Decomposing this interaction indicates that the interaction is driven by a clear perceived intelligence-humanness link for White targets, *β* = 0.29, *t* = 4.14, *p* < .001, an effect that is eliminated for Black targets, *β* = 0.05, *t* = 1.14, *p* = .255, see Table 2.

**Table 1:** Unstandardized coefficients of attractiveness, intelligence, target gender, and target race on humanness.

Predictor	*b*	*t*	*p*
**Attractiveness**	**0.42**	**9.68**	**<.001**
**Perceived Intelligence**	**0.17**	**4.10**	**<.001**
**Gender**	**0.14**	**5.26**	**<.001**
**Race**	**0.53**	**20.14**	**<.001**
**Race x Gender**	**−0.23**	**−8.81**	**<.001**
Gender x Attr	−0.01	−0.27	.786
Gender x Int	−0.02	−0.44	.658
Race x Attr	−0.04	0.82	.415
**Race x Int**	**−0.12**	**−2.84**	**.004**
Gender x Race x Attr	0.03	0.69	.498
Gender x Race x Int	0.03	0.64	.521

*Note*: Race and gender were coded such that White = −1, Black = 1, Male = −1, Female = 1. Attractiveness, perceived intelligence, and ascriptions of humanness were Z-transformed before being input into model. Analyses were conducted using the *lm* function in R, thus performing an OLS regression.

**Table 2:** Interaction between target race and perceived intelligence in humanness ratings.

Target Group	Predictor	*b*	*t*	*p*
Black Targets	**Constant**	**0.54**	**18.73**	**<.001**
	**Attractiveness**	**0.38**	**6.79**	**<.001**
	Perceived Intelligence	0.05	1.14	.254
	**Gender**	**−0.09**	**−3.27**	**.001**
	Gender x Attr	0.02	0.31	.746
	Gender x Int	0.01	0.18	.856
White Targets	**Constant**	**−0.53**	**−11.72**	**<.001**
	**Attractiveness**	**0.46**	**8.00**	**<.001**
	**Perceived Intelligence**	**0.29**	**4.144**	**<.001**
	**Gender**	**0.37**	**8.27**	**<.001**
	Gender x Attr	−0.04	−0.72	.471
	Gender x Int	−0.04	−0.65	.518

*Note*: Gender was coded such that Male = −1, Female = 1. Attractiveness, perceived intelligence, and ascriptions of humanness were Z-transformed before being input into model. Analyses were conducted using the *lm* function in R, thus performing an OLS regression.

As racial priming has been seen to produce stereotype consistent responses in both Black and White participants, we provide exploratory analyses by participant race in the supplemental materials [28].

In sum, we replicated Alaei and colleagues’ [12] most central findings that facial attractiveness and perceived intelligence are strong predictors of dehumanization of White targets. However, we did not find that attractiveness and intelligence differentially predicted humanness for male and female faces (i.e., gender x attractiveness or a gender x intelligence interactions). Notably though, our stimuli differed from Alaei and colleagues’ [12] in multiple ways. Perhaps most centrally, whereas Alaei used faces belonging to college students, our study utilized the more age and race diverse stimuli from the Chicago Face Database. Thus, it could be that their finding that perceived intelligence is a stronger predictor of humanness than perceived attractiveness is population specific.

More importantly, we found novel empirical evidence that people use different dimensions to infer humanness across Black and White targets. Whereas attractiveness was linked to humanness for both Black and White faces, perceivers only appeared to weight perceived intelligence in humanness judgments of White faces. This is important for multiple reasons. First, whereas past work has shown that people weigh intelligence more for some groups than others, this is the first work to show that apparent intelligence may be devalued (at least in its use for inferring humanness) for Black faces, making this a clear departure from past work. Second, this finding is the first to our knowledge that investigates how people make dehumanizing inferences based on facial features differentially (and in the case of attractiveness, similarly) across race. Finally, this finding is important because it connects conceptually to the deep well of past work indicating that the treatment of Black Americans is often based on physicalized stereotypes.

### Study 2

In Study 2 we aimed to replicate and extend our findings from Study 1. One issue with Study 1 is that participants rated targets in a mono-categorical context. That is, they rated only one target race x target gender category in the rating task (i.e., either White men, White women, Black men, or Black women). This methodological choice was intentional because it mapped closely onto the procedure of Alaei and colleagues [12] in which participants rated either White men or White women. However, it is unclear whether identical effects would occur if participants made their ratings of faces in a multi-racial context.

The context in which perceivers evaluate target race x target gender intersectional targets can affect stereotype salience, regulation, and application. Indeed, Petsko and Bodenhausen’s [29] Lens Model of intersectional stereotyping argues that the available variability in categories and social contexts may change what categories and intergroup motives are activated and applied. Of interest in Study 2 was whether the attractiveness-humanness and perceived intelligence-humanness relationships observed in Study 1 and in past research would change when participants made their judgments in a *multi*-category rather than in a mono-category context. Thus, in Study 2, participants again rated a single dimension (attractiveness, perceived intelligence, or humanness) but for a subset of faces including all four target race x target gender intersections in the stimulus set (rather than for a subset of faces in a single race x gender intersection, as in Study 1 and in Alaei and colleagues [12]).

Despite this past work indicating that an intergroup context can change the evaluation of dimensions, it is less clear from this model precisely how the dimensions of evaluation would change. One possibility is that a multi-categorical evaluative space could amplify the effects of the categories. Indeed, perhaps when people see all four target race x target gender intersectional categories, then the categories themselves may become particularly salient and thus likely to affect judgment. Alternately, we thought it more likely that the multi-categorical space may make egalitarian motives particularly salient, and in that context may dampen participants’ tendencies to utilize different dimensions to evaluate people across groups. To our knowledge this is the first study to investigate how the relationship between dimensions of face evaluation may change based on category presentation [14,15]. Because multiple predictions seemed sensible and no prior work had tested these questions, we approached Study 2 in an exploratory manner.

### Method

#### Participants.

Participants (*n* = 227) were mTurk workers paid $3.62 for a 30-minute study. 35 were excluded for having given homogeneous responses or not completing the study, and 2 were removed for not reporting to have taken the study seriously, resulting in an analytic sample of *n* = 190. Participants recruited were currently residing within the United States and fluent in English. 42.6% of participants identified as female, and 57.4% as male, with a mean age of 39.70 years old (*SD* = 11.37). Participants primarily identified as White (66.3%), but a notable portion identified as Black or African American (15.8%) or reported multiple racial identities (5.8%). Many participants identified as slightly to very liberal in political identity 53.7%, with 14.7% identifying as moderate, and 31.0% identifying as slightly to very conservative (1 participant did not report a political ideology).

### Stimuli and Measures

The CFD stimuli used in Study 1 were used in Study 2, and measures of humanness ascriptions and perceived traits were identical to Study 1 [24].

### Procedure

A stimuli-with-block design was used to reduce participant burden while retaining a large number of stimuli to ensure sufficient power [27]. Three independent conditions were created consisting of 120 unique faces. Each condition was composed of equal numbers of each race-gender category (30 Black women, 30 Black men, 30 White women, 30 White men per condition). Random number generators were used to assign each stimuli to condition, ensuring that no stimuli appeared in more than one condition.

Participants were randomly assigned to condition (i.e., assigned to see a specific subset of stimuli), and within each condition, participants were randomly assigned to rate the faces within their condition on a single trait (i.e., either attractiveness, intelligence, or humanness).

Participants thus rated all 120 faces of varying races and genders on a single trait. Analyses mirrored Study 1 protocol.

## Results and discussion

Of interest in Study 2 was whether the findings of Study 1 replicate when the race and gender of stimuli were intermixed during the rating phase. To test this, we employed the identical analyses as in Study 1, which indicated the overall model accounted for significant variance in humanness ratings, *F*(11, 348) = 38.44, *p* < .001, *R*^*2*^ = .53.

Conceptually replicating both Alaei and colleagues and Study 1, we again found that facial attractiveness and perceived intelligence were significant predictors of ascriptions of humanness; see Table 3. We again found a target race x perceived intelligence interaction, such that the perceived intelligence-humanness link observed only for White targets, *β* = 0.25, *t* = 4.08, *p* < .001, but not for Black targets, *β* = −0.0009, *t* = −0.009, *p* = .993, when accounting for attractiveness in the model (see Table 4). This pattern closely replicates the findings of Study 1. Unlike Study 1, we no longer see a main effect of gender on humanness (*p* > .05).

**Table 3:** Unstandardized coefficients of attractiveness, intelligence, target gender, and target race on humanness.

Predictor	*b*	*t*	*p*
**Attractiveness**	**0.53**	**4.81**	**<.001**
**Perceived Intelligence**	**0.27**	**2.68**	**.008**
Gender	−0.03	−0.29	.769
**Race**	**−0.48**	**−4.32**	**<.001**
Race x Gender	−0.20	−1.26	.207
Gender x Attr	−0.11	−0.72	.472
Gender x Int	−0.03	−0.22	.823
Race x Attr	0.31	1.75	.081
**Race x Int**	**−0.35**	**−2.23**	**.027**
Gender x Race x Attr	−0.24	−0.98	.330
Gender x Race x Int	0.20	0.90	.367

*Note*: Race and gender were coded such that White = −1, Black = 1, Male = −1, Female = 1. Analyses were conducted using the *lm* function in R, thus performing an OLS regression.

**Table 4:** Interaction between target race and perceived intelligence in humanness ratings.

Target Group	Predictor	*b*	*t*	*p*
Black Targets	**Constant**	**−0.26**	**−4.16**	**<.001**
	**Attractiveness**	**0.67**	**6.21**	**<.001**
	Perceived Intelligence	−0.001	−0.01	.992
	Gender	−0.11	−1.84	.068
	Gender x Attr	−0.17	−1.59	.115
	Gender x Int	0.08	0.88	.382
White Targets	**Constant**	**0.32**	**6.88**	**<.001**
	**Attractiveness**	**0.47**	**7.56**	**<.001**
	**Perceived** **Intelligence**	**0.25**	**4.08**	**<.001**
	Gender	−0.02	−0.35	.725
	Gender x Attr	−0.02	−0.27	.789
	Gender x Int	−0.05	−0.86	.389

*Note*: Gender was coded such that Male = −1, Female = 1. Analyses were conducted using the *lm* function in R, thus performing an OLS regression.

## General Discussion

Our goal in the present research was to conceptually replicate research linking perceptual cues of attractiveness and perceived intelligence to humanness in White male and female faces and to characterize whether these effects occur amongst Black male and female faces [12]. We see a consistent tendency for attractiveness to drive judgments of humanness across categories and contexts. Indeed, the attractiveness-humanness link was observed in every case here and in the original Alaei and colleagues’ work. Second, and novel to the current work, we also see a consistent tendency for the perceived intelligence-humanness link to be qualified by target race. Whereas participants appear to link perceived intelligence with ascriptions of humanness for White targets, this does not appear to be true whatsoever for Black targets. Presenting the stimuli in a multi-racial, multi-gender context removed the degree that gender, and a gender by race interaction predicts dehumanization.

This research notably expands on the face perception and dehumanization literatures. While previous work has looked at group differences in trait perceptions or dehumanization or at how facial features correlate with trait judgements in White male faces [15,30], the present work described not only the perceptual level cues used to predict blatant dehumanization, but also shows that these perceptual cues are *differentially weighted* depending on the race on the target. Classic work in face perception has shown that perceivers use structural features about the face to infer traits [30], and that these perceived traits can become correlated within a participant’s mind [14], but it is only recently that the importance of target group membership and stereotypes has been discovered. Indeed, Xie and colleagues [15] found that perceiver stereotypes predicted the structure of their facial impressions, creating a strong argument for not only looking at the predictors of trait inferences, but also investigating how these relationships are moderated by target group. The present study not only points to the underlying impressions that predict dehumanization at first impressions, but also highlights how different trait inferences are used to infer the humanness of targets.

The focus on blatant dehumanization is particularly critical given the many negative outcomes associated with being ascribed less humanity. Leadership racial selection bias can be predicted by the uniquely human skills the job requires [31]. Judgements relating to use of police force against a target were found to be predicted by target humanization through ascriptions of self-control and denial of pain [17]. Individuals convicted of capital crimes who are described in apelike (i.e., animalistic dehumanization) in the news were found to be more likely to be executed by the state than convicted individuals not described as apelike [13]. Thus, understanding not only the mechanism for predicting dehumanization from first impressions, but also understanding how these relationships may differ by groups, aids in identifying those most vulnerable to being dehumanized and experiencing the negative consequences of being seen as less human.

Several limitations offer exciting avenues for future research and help contextualize our findings in the broader literature. First, despite the variety and sample size of our facial stimuli within groups, dehumanization can occur differently in racial groups other than Black and White Americans [31]. Whereas Black individuals are often dementalized, groups stereotyped as high intelligence such as East Asian targets may show different relationships between perceived traits and dehumanization [18]. Thus, research should consider how these effects may vary across more racial groups. Additionally, both facial affect and denial of complex emotions are implicated in the process of dehumanization, meaning that samples of faces varying in emotional expressions could yield results beyond our findings using neutral expressions [18,32,33]. Second, our participants only rated faces on a single trait to avoid the possibility of participants artificially correlating these traits during the experiment. Although this procedure protects against the potential of artificial associations, and against demand effects essentially guiding participants to use one trait to infer another, it prevents examination of participant level effects. Importantly, work on racial priming as found that within group variation, such as how prototypically “Black” a face is, influences how the face is evaluated, necessitating individual stimulus level analyses [34]. Notably, the strength of almost all effects, as exampled by the *T* statistic, dropped from Study 1 to Study 2. This cannot be attributed to power, or stimuli various, as the sample stimuli are used in both studies, and as stimuli are the level of analysis both studies are comparably powered. Thus, we infer that the presentation of the stimuli as intermixed, compared to separate by racial gender group drove this drop in predictive strength in Study 2. Indeed, gender, and thus gender by race interaction, may have been less saliant in an interracial context compared to a single racial context. Additionally, given the stimuli level of analysis aggregating rates across participants within stimuli, these stimuli values are subject to sample specific variation. Further work should investigate these questions within a methodology that allows for multilevel analyses. Indeed, prior work has found assessments of attractiveness to occur prior to assessments of competence, indicating that within perceiver measurement is necessary to uncover the mechanism of these effects [35]. Exploratory analyses investigating the role of participant race is included in the SOM. Third, it is important to note that, due to experimental error, the Black female condition in our Study 1 a small error in the instructions, which led us to re-collect this condition. This means that participants to this condition were not randomly assigned, leaving open the possibility of history effects. That said, it is important to note that many of the same core findings occurred in both studies, somewhat attenuating the magnitude of the concern. Lastly, the present work does not characterize the higher-dimensional trait space that may occur beyond the three traits measured, indicating that our observed effects may themselves be mediated by other trait judgments. For example, attractiveness and perceived intelligence are both strongly and positively related to trustworthiness judgements which is differently related to dehumanization of White and Black faces [36]. Thus, future research would benefit from a greater understanding of this higher-dimensional trait space [37].

In conclusion, we found that perceptual factors are predictive of (de)humanization of individual faces differently across race. Whereas perceived attractiveness is a strong and consistent predictor of (de)humanization, perceived intelligence was only a predictor for White targets. Thus, the visual cues used to ascribe humanness, differ across intersectional categories (see Xie et al., [15] for similar effects with other traits), indicating both that trait-humanness links are bounded by social categorization.

## Supporting information

S1 File Supplemental analyses. (DOCX)
