# Supplementary material for: Intersectional dehumanization in faces: Facial attractiveness and perceived intelligence differentially predict dehumanization across intersecting race and gender identities
Source: PLoS One. 2026 Jul 1;21(7):e0351086. doi: 10.1371/journal.pone.0351086 (PMC13322522; doi:10.1371/journal.pone.0351086)
Supplement: S1 File — (DOCX) [file pone.0351086.s001.docx]

**Supplemental Table 1**

*Study 1 Correlations by Target Race*

|  |  | Attractiveness | Perceived Intelligence | Humanness Ascription |
| --- | --- | --- | --- | --- |
| Black | Attractiveness | 1 |  |  |
|  | Perceived Intelligence | .80_q_ | 1 |  |
|  | Humanness Ascription | .69_b_ | .57_c_ | 1 |
| White | Attractiveness | 1 |  |  |
|  | Perceived Intelligence | .73_a_ | 1 |  |
|  | Humanness Ascription | .72_d_ | .73_a_ | 1 |

Subscripts used to denote statistical difference (*p* < .05) on Fisher’s R to Z transformation. Unique subscripts indicate statistical difference between two values.

**Supplemental Table 2**

*Study 1 Correlations by Target Race and Target Gender*

|  |  | Attractiveness | Perceived Intelligence | Humanness Ascription |
| --- | --- | --- | --- | --- |
| Black Men | Attractiveness | 1 |  |  |
|  | Perceived Intelligence | .77_a_ | 1 |  |
|  | Humanness Ascription | .66 _b_ | .55 _b_ | 1 |
| Black Women | Attractiveness | 1 |  |  |
|  | Perceived Intelligence | .83 _a_ | 1 |  |
|  | Humanness Ascription | .74 _a,b_ | .65 _b_ | 1 |
| White Men | Attractiveness | 1 |  |  |
|  | Perceived Intelligence | .75 _a_ | 1 |  |
|  | Humanness Ascription | .74 _a,b_ | .70 _a,b_ | 1 |
| White women | Attractiveness | 1 |  |  |
|  | Perceived Intelligence | .73 _a,b_ | 1 |  |
|  | Humanness Ascription | .80 _a_ | .70 _a,b_ | 1 |

Subscripts used to denote statistical difference (*p* < .05) on Fisher’s R to Z transformation. Unique subscripts indicate statistical difference between two values.

**Supplemental Table 3**

*Predictors of Humanness Ascriptions by Participant Race Study 1*

| White Participants | | | | POC Participants | | | |
| --- | --- | --- | --- | --- | --- | --- | --- |
| Predictor | Beta | *t* | *p* | Predictor | Beta | *t* | *p* |
| **Attractiveness** | **0.42** | **10.22** | **<.001** | **Attractiveness** | **0.33** | **6.63** | **<.001** |
| **Perceived Intelligence** | **0.17** | **4.14** | **<.001** | Perceived Intelligence | 0.10 | 2.15 | 0.32 |
| **Gender** | **0.21** | **4.14** | **<.001** | **Gender** | **-0.25** | **-5.16** | **<.001** |
| **Race** | **0.56** | **20.52** | **<.001** | **Race** | **0.48** | **9.99** | **<.001** |
| **Race x Gender** | **-0.16** | **-6.00** | **<.001** | **Race x Gender** | **0.17** | **3.51** | **<.001** |
| Gender x Attr | -0.01 | -0.21 | .831 | **Gender x Attr** | **0.10** | **2.03** | **.043** |
| Gender x Int | -0.05 | -1.16 | .237 | Gender x Int | -0.03 | -0.62 | .539 |
| Race x Attr | -0.03 | -0.73 | .464 | Race x Attr | -0.10 | -1.96 | 0.05 |
| **Race x Int** | **-0.13** | **-3.22** | **.004** | Race x Int | 0.03 | 0.74 | .459 |
| Gender x Race x Attr | 0.02 | 0.54 | .590 | Gender x Race x Attr | 0.05 | 0.98 | .326 |
| Gender x Race x Int | 0.03 | 0.70 | .486 | **Gender x Race x Int** | **0.11** | **2.51** | **.012** |

*Note*: Race and Gender were coded such that White = -1, Black = 1, Male = -1, Female = 1.

**Supplemental Table 4**

*Study 1 Correlations by Target Race and Target Gender, POC Participants*

|  |  | Attractiveness | Perceived Intelligence | Humanness Ascription |
| --- | --- | --- | --- | --- |
| Black Men | Attractiveness | 1 |  |  |
|  | Perceived Intelligence | .54 | 1 |  |
|  | Humanness Ascription | .17 | .15 | 1 |
| Black Women | Attractiveness | 1 |  |  |
|  | Perceived Intelligence | .55 | 1 |  |
|  | Humanness Ascription | .62 | .55 | 1 |
| White Men | Attractiveness | 1 |  |  |
|  | Perceived Intelligence | .46 | 1 |  |
|  | Humanness Ascription | .52 | .41 | 1 |
| White women | Attractiveness | 1 |  |  |
|  | Perceived Intelligence | .41 | 1 |  |
|  | Humanness Ascription | .42 | .11 | 1 |

POC participants refers to “people of color” participants, which we operationalized as any participant that did not identify as mono-racial White.

**Supplemental Table 5**

*Study 1 Correlations by Target Race and Target Gender, White Participants*

|  |  | Attractiveness | Perceived Intelligence | Humanness Ascription |
| --- | --- | --- | --- | --- |
| Black Men | Attractiveness | 1 |  |  |
|  | Perceived Intelligence | .77 | 1 |  |
|  | Humanness Ascription | .68 | .57 | 1 |
| Black Women | Attractiveness | 1 |  |  |
|  | Perceived Intelligence | .82 | 1 |  |
|  | Humanness Ascription | .74 | .62 | 1 |
| White Men | Attractiveness | 1 |  |  |
|  | Perceived Intelligence | .73 | 1 |  |
|  | Humanness Ascription | .75 | .70 | 1 |
| White women | Attractiveness | 1 |  |  |
|  | Perceived Intelligence | .66 | 1 |  |
|  | Humanness Ascription | .80 | .65 | 1 |

**Supplemental Table 6**

*Study 2 Correlations by Target Race*

|  |  | Attractiveness | Perceived Intelligence | Humanness Ascription |
| --- | --- | --- | --- | --- |
| Black | Attractiveness | 1 |  |  |
|  | Perceived Intelligence | .79_a_ | 1 |  |
|  | Humanness Ascription | .59 _b_ | .48 _b,c_ | 1 |
| White | Attractiveness | 1 |  |  |
|  | Perceived Intelligence | .71 _a,d_ | 1 |  |
|  | Humanness Ascription | .74 _a,d_ | .67 _b,d_ | 1 |

Subscripts used to denote statistical difference (*p* < .05) on Fisher’s R to Z transformation. Unique subscripts indicate statistical difference between two values.

**Supplemental Table 7**

*Study 2 Correlations by Target Race and Target Gender*

|  |  | Attractiveness | Perceived Intelligence | Humanness Ascription |
| --- | --- | --- | --- | --- |
| Black Men | Attractiveness | 1 |  |  |
|  | Perceived Intelligence | .77 _a_ | 1 |  |
|  | Humanness Ascription | .60 _b_ | .43 _c_ | 1 |
| Black Women | Attractiveness | 1 |  |  |
|  | Perceived Intelligence | .81 _a_ | 1 |  |
|  | Humanness Ascription | .61 _b_ | .53 _b,c_ | 1 |
| White Men | Attractiveness | 1 |  |  |
|  | Perceived Intelligence | .60 _b_ | 1 |  |
|  | Humanness Ascription | .71 | .61 _b_ | 1 |
| White women | Attractiveness | 1 |  |  |
|  | Perceived Intelligence | .79 _a_ | 1 |  |
|  | Humanness Ascription | .74 _a,d_ | .70 _b,d_ | 1 |

**Supplemental Table 8**

*Predictors of Humanness Ascriptions by Participant Race Study 2*

| White Participants | | | | POC Participants | | | |
| --- | --- | --- | --- | --- | --- | --- | --- |
| Predictor | Beta | *t* | *p* | Predictor | Beta | *t* | *p* |
| **Attractiveness** | **0.45** | **3.98** | **<.001** | **Attractiveness** | **0.34** | **3.03** | **.003** |
| **Perceived Intelligence** | **0.35** | **3.41** | **<.001** | Perceived Intelligence | 0.19 | 1.77 | .077 |
| Gender | -0.05 | -.43 | .667 | Gender | 0.20 | 1.53 | .128 |
| Race | -0.20 | -1.70 | .090 | **Race** | **-0.36** | **-2.75** | **.006** |
| Race x Gender | -0.17 | -1.04 | .301 | Race x Gender | -0.29 | -1.58 | .114 |
| Gender x Attr | 0.00 | -0.01 | .996 | Gender x Attr | 0.04 | 0.27 | .791 |
| Gender x Int | -0.12 | -0.75 | .451 | Gender x Int | -0.09 | -0.58 | .564 |
| Race x Attr | 0.30 | 1.58 | .115 | Race x Attr | 0.10 | 0.62 | .533 |
| Race x Int | -0.28 | -1.65 | .100 | **Race x Int** | **-0.34** | **-2.16** | **.031** |
| Gender x Race x Attr | 0.28 | 1.17 | .245 | Gender x Race x Attr | 0.10 | 0.47 | .639 |
| Gender x Race x Int | -0053 | -1.37 | .172 | Gender x Race x Int | 0.12 | 0.57 | .568 |

*Note*: Race and gender were coded such that White = -1, Black = 1, Male = -1, Female = 1.

**Supplemental Table 9**

*Study 2 Correlations by Target Race and Target Gender, POC Participants*

|  |  | Attractiveness | Perceived Intelligence | Humanness Ascription |
| --- | --- | --- | --- | --- |
| Black Men | Attractiveness | 1 |  |  |
|  | Perceived Intelligence | .57 | 1 |  |
|  | Humanness Ascription | .28 | .07 | 1 |
| Black Women | Attractiveness | 1 |  |  |
|  | Perceived Intelligence | .55 | 1 |  |
|  | Humanness Ascription | .52 | .21 | 1 |
| White Men | Attractiveness | 1 |  |  |
|  | Perceived Intelligence | .45 | 1 |  |
|  | Humanness Ascription | .48 | .39 | 1 |
| White women | Attractiveness | 1 |  |  |
|  | Perceived Intelligence | .64 | 1 |  |
|  | Humanness Ascription | .61 | .47 | 1 |

POC participants refers to “people of color” participants, which we operationalized as any participant that did not identify as mono-racial White.

**Supplemental Table 10**

*Study 2 Correlations by Target Race and Target Gender, White Participants*

|  |  | Attractiveness | Perceived Intelligence | Humanness Ascription |
| --- | --- | --- | --- | --- |
| Black Men | Attractiveness | 1 |  |  |
|  | Perceived Intelligence | .78 | 1 |  |
|  | Humanness Ascription | .65 | .53 | 1 |
| Black Women | Attractiveness | 1 |  |  |
|  | Perceived Intelligence | .82 | 1 |  |
|  | Humanness Ascription | .60 | .58 | 1 |
| White Men | Attractiveness | 1 |  |  |
|  | Perceived Intelligence | .57 | 1 |  |
|  | Humanness Ascription | .59 | .57 | 1 |
| White women | Attractiveness | 1 |  |  |
|  | Perceived Intelligence | .79 | 1 |  |
|  | Humanness Ascription | .74 | .68 | 1 |
